# 肺癌筛查方法现状

**DOI:** 10.3779/j.issn.1009-3419.2016.10.14

**Published:** 2016-10-20

**Affiliations:** 101149 北京，首都医科大学附属北京胸科医院/北京市结核病胸部肿瘤研究所肿瘤内科 Department of Medical Oncology, Beijing Chest Hospital, Capital Medical University/Beijing Tuberculosis and Toracic Tumor Research Institute, Beijing 101149, China

**Keywords:** 肺肿瘤, 筛查, 低剂量计算机断层扫描, 循环肿瘤细胞, 自身抗体, Lung neoplasms, Screening, Low-dose computed tomography, Circulating tumor cells, Autoantibodies

## Abstract

肺癌是目前恶性肿瘤死亡的首要原因，早期诊断对肺癌的预后至关重要。研究显示低剂量计算机断层扫描（computed tomography, CT）筛查可以使肺癌的死亡率下降。但其存在的问题不可忽视，如过高的假阳性率、过度诊断、辐射效应等。作为一种肿瘤无创筛查方法，血液相关肿瘤标志物的检测，在肺癌早期诊断中显示出良好的敏感性和特异性。如何利用现有的筛查手段，建立肺癌筛查综合模式，需要更多大规模的临床研究。

## Abstract (en)

Lung cancer is the leading cause of cancer mortality currently. Early diagnosis is crucial to the good prognosis of lung cancer. Evidence has shown that low-dose computed tomography (CT) screening can make the decline in the mortality of lung cancer. However, there are still many problems in the screening, such as, high false-positive rates, overdiagnosis and radiation exposure. As another non-invasive tumor screening method, blood-based tumor markers showed a high sensitivity and specificity in early diagnosis of lung cancer in recent years. How to establish a comprehensive mode for lung cancer screening by use existing screening methods, we need more clinical research.

当今，肺癌是恶性肿瘤相关死亡的首要原因^[1]^。流行病学资料^[2]^显示，2012年全球肺癌死亡病例数约为160万，占全部恶性肿瘤死亡的19.4%。在我国新近公布的统计数据中，我国2015年肺癌新发病例约73万，死亡病例约61万，发病率及死亡率均已成为恶性肿瘤首位^[3]^。尽管近年来肺癌治疗手段有较快发展，然而总体预后并无明显改善，目前5年总生存率仅为16%-18%^[4]^。究其原因是缺乏有效的早期诊断方法，肺癌确诊时70%-80%均为中晚期。Ⅰ期肺癌5年存活率达60%-70%，而Ⅳ期肺癌存活率不到5%^[3]^。因此肺癌的早期筛查诊断是减少其死亡率及延长生存率的关键因素。但如何进行合理有效的肺癌早期筛查，对高危人群进行有效监控是目前面临的一个重要问题，本文就目前肺癌筛查方法问题作一简单综述。

## 1 肺癌早期筛查现状

自20世纪90年代开始，随着胸部低剂量计算机断层扫描（low-dose computed tomography, LDCT）技术的发展，肺癌筛查研究进入LDCT时代，并迅速成为肺癌早期筛查的热点。目前全球多个国家开展了LDCT肺癌筛查研究，其中国际早期肺癌行动计划（International Early Lung Cancer Program, I-ELCAP）^[5, 6]^、美国国家癌症研究所（National Cancer Institute, NCI）发起的大型肺癌筛查随机对照研究——国家肺癌筛查试验（National Lung Screening Trial, NLST）^[7, 8]^、荷兰-比利时的多中心随机对照研究项目（Dutch-Belgian Randomized Lung Cancer Screening Trial, NELSON）^[9, 10]^、意大利的LDCT肺癌筛查的随机对照研究项目（ITALUNG和DANTE）^[11, 12]^等均为全球较为著名的肺癌筛查研究项目。这些结果明确提示LDCT肺癌筛查具有非常显著的临床意义。不仅可以检出更多更早期的肺癌，降低肺癌死亡率，改善肺癌患者预后；而且能提高生活质量，包括减少肺癌相关症状负担、减少治疗相关并发症、提高戒烟率；同时检出其他需要治疗的疾病。基于这些令人振奋的结果，美国国家综合癌症网络（National Comprehensive Cancer Network, NCCN）、美国胸外科协会（American Association for Thoracic Surgery, AATS）、美国胸科医师学院（American College of Chest Physicians, ACCP）、美国临床肿瘤协会（American Society of Clinical Oncology, ASCO）、美国癌症协会（American Cancer Society, ACS）、美国预防服务工作组（U.S. Preventive Services Task Force, USPSTF）等多家美国权威医学组织于2011年起陆续推出了肺癌筛查指南，推荐在高危人群中进行LDCT肺癌筛查。我国的LDCT肺癌筛查工作也在加速推进^[13]^。2012年启动的国家重大公共卫生专项-城市癌症早诊早治项目，针对我国城市高发的5大癌症（肺癌、大肠癌、上消化道癌、乳腺癌和肝癌）进行危险因素调查、高危人群评估、癌症筛查和卫生经济学研究，目前已在全国16个省份开展了LDCT肺癌筛查，预计最终肺癌筛查人数超过20万，将是迄今全球最大规模的LDCT肺癌筛查项目。同时在中华医学会放射学分会心胸学组多位专家的共同努力下，我国首版“低剂量螺旋CT肺癌筛查专家共识”于2015年出台^[14]^。

## 2 肺癌早期筛查人群的确定

连续进行LDCT肺癌筛查会增加辐射风险和经济负担，为了保证筛查者的获益，肺癌筛查应该在高危人群中进行。合理、准确地选择筛查对象，可降低无效筛查比例，提高肺癌筛查的卫生经济学效益。在不同的指南中，高危人群的定义不尽相同。NCCN指南^[15]^将高危人群定义为：年龄55岁-74岁，吸烟≥30包年（并且戒烟＜15年）；或者年龄≥50岁，吸烟≥20包/年，且合并上述另一项危险因素（不包括被动吸烟）。USPSTF推荐年龄在55岁-80岁之间；身体症状不明显；烟龄超过30年，且每年至少30包；成功戒烟时间不超过15年的吸烟者需要每年定期接受LDCT筛查。我国的肺癌危险因素与西方发达国家不尽相同。随着工业化、城市化进程的加快，我国已成为世界上大气污染较严重的国家之一，因此大气污染暴露是一个不容忽视的危险因素。另外，对于女性被动吸烟和厨房油烟等危险因素也需予以足够重视。目前我国中华医学放射学分会最新推出的“LDCT肺癌筛查专家共识”^[14]^建议将高危人群定义为：①年龄50岁-75岁；②至少合并以下一项危险因素：吸烟≥20包年，其中包括戒烟时间不足15年者；被动吸烟者；有职业暴露史（石棉、铍、铀、氡等接触者）；有恶性肿瘤病史或肺癌家族史；有慢性阻塞性肺病（chronic obstructive pulmonary diseases, COPD）或弥漫性肺纤维化病史。

目前，各个筛查指南对高危人群的界定都不尽相同，但大多评价标准都局限于吸烟人群，而忽视了非吸烟高危人群，并且缺乏对肺癌高危因素的量化评估。利物浦肺计划（Liverpool Lung Project, LLP）风险模型^[16]^的出现为肺癌高危人群的选择提供了新的思路。利用*Logistic*回归模型估算高危人群5年内肺癌的发生率。其中所确定的危险因素有肺癌家族史、粉尘接触史、肺炎史、除肺癌外的恶性肿瘤史和吸烟年限。但LLP实验为匹配病例对照实验，无法估计年龄性别相关的危险因素，在一定程度上影响了结果的准确性，使其可靠性相对薄弱。PLCO（Prostate, Lung, Colorectal, and Ovarian）模型^[17]^被用来与NLST的高危人群标准作比较。结果显示，PLCO模型的真阳性率为4.0%，敏感度为83.0%，而NLST分别为3.2%和71.1%，肺癌的检出率提高了0.8%。在近期*JAMA*杂志上，Katki等^[18]^着重强调了一种筛选LDCT筛查人群的增强型风险评估模型较传统USPSTF推荐方案所体现出来的巨大优势。该研究是基于PLCO对照组在相关数据，建立肺癌发病率和死亡率的风险经验模型。按照USPSTF推荐方案，约900万美国烟民宜行肺癌筛查，而且5年内可有效避免约46, 488例死亡患者，而采用新的风险筛选模型，宜行肺癌筛查的烟民数量与前者相似，约为900万，但在有效避免患者死亡上有明显优势，可避免不低于20%（约55, 717例）。在预测准确性上，新的风险模型较USPSTF推荐方案可降低17%的必要筛查数量（即避免1例肺癌死亡需要进行筛查的人数）。新的肺癌筛查风险模型的出现，为高危人群的选择和确定提出了新的方向，但不能忽视的是，大部分接受肺癌LDCT筛查者并没有从中获益。对于临床医生来说，患者个体接受癌症筛查是否为最优策略，仍是一个挑战。

## 3 肺癌早期筛查的方法

### 3.1 LDCT筛查

目前研究的最多也最为大家公认的就是LDCT肺癌筛查。I-ELCAP研究^[5, 6]^对31, 567例肺癌高危人群每年进行LDCT检查，发现LDCT肺癌检出率是胸部X线的4倍以上，≥40岁人群中首次LDCT发现肺癌的阳性率为1.3%，年度检查的阳性率为0.3%；≥60岁人群中首次LDCT和年度检查发现肺癌的阳性率则分别为2.7%和0.6%。该成果显示每年定期LDCT筛查发现的肺癌病例80%以上为Ⅰ期，肺癌预期总10年生存率80%（不论临床分期和接受何种治疗）；若及时手术，预期总10年生存率高达92%。2002年开始的NLST研究^[7, 8]^是第一个具有足够统计功效并且实施良好的多中心随机对照研究，入组53, 454例患者，年龄55岁-74岁，均为重度吸烟者≥30包年（或者戒烟≤15年）。其研究结果显示，与胸部X线比较，对高危人群进行每年1次连续3年的LDCT筛查可使肺癌死亡率下降20.3%（*P*=0.004），全因死亡率下降了6.7%（*P*=0.02）。这是第一项LDCT筛查可以降低肺癌病死率的随机对照试验，提供了机有说服力的证据。但是同时LDCT筛查早期肺癌是否能使患者真正受益一直存在争议，其中主要问题是筛查过度、诊断的误漏、临床随访时间的长期性、医疗费用的增加和辐射剂量诱发癌变的可能性等。在进行筛查时的基线检查中，有27%会出现阳性结果，但96%为非肺癌。NLST研究中^[7, 8]^，阳性发现在LDCT组和胸部X线组分别为39.1%和16.0%，而非肺癌阳性率分别是96.4%和94.5%。总研究人数中5.5%的患者进行了追加PET/CT检查；1.5%的患者进行了非手术有创检查，其中73%为良性结节；2.6%的患者进行了手术，其中24%为良性结节。而且在NLST后续检查中，1.2%的患者没有发现肺癌而进行了有创检查，0.7%的患者没有肺癌而进行了胸腔镜、纵隔镜和开胸检查；对于良性结节进行有创检查也较常见，NLST中达到了73%；有创检查后60天内死亡人数分别是LDCT组16例（10例为肺癌），胸部X线组10例（全部为肺癌）。这些结果强烈警示我们应该进行更深入的研究，建立更高效的筛查模式。

### 3.2 血液分子标志物的检测

LDCT肺癌筛查的风险与获益已经让我们意识到没有一种筛查或诊断方法能够准确鉴别所有肺癌，因此对肺癌的早期筛查与诊断需要建立一种综合模式。运用其他诊断手段联合LDCT筛查成为关键。联合筛查手段应具备以下特点：①无创；②有较好的量化指标和可重复性；③较高的敏感性和特异性。血液分子标志物通过检查人体肿瘤分子生物层面的改变来发现肿瘤，同时具备无创、简单、便捷的特点，对肺癌早期筛查和诊断也具有重要意义。

#### 3.2.1 小分子RNA（microRNAs）

基于蛋白的传统肿瘤标志物如癌胚抗原（carcino-embryonic antigen, CEA）、血清鳞状细胞抗原（serum squamous cell carcinoma antigen, SCC-Ag）等对早期肺癌的敏感性低于10% ^[19]^，而且区分肺癌与良性肺病的特异性低。miRNAs是生物体内的一种非编码RNA，在肿瘤患者体内某些microRNAs会有显著变化^[20]^。因此通过外周血定量检测microRNAs成为肺癌早期诊断的新焦点。Sozzi等^[21]^采用基于意大利多中心肺癌检测（Multicenter Italian Lung Detection, MILD）的血清miRNAs检测，对参与MILD实验者留取的血浆标本进行检测24种miRNA组成信号分类（miRNA signature classifier, MSC），根据其表达分为高危、中危和低危三组，对肺癌诊断的敏感性和特异性分别为87%和81%，对诊断的阴性预测值为99%，对死亡的预测值达到99.86%；在LDCT组的假阳性率为19.4%，加入MSC后，其敏感度下降到69%，但假阳性率下降至3.7%。因此在LDCT肺癌筛查的同时参考MSC的结果可以降低假阳性率，但MSC未对不同病理类型分别检测，在一定程度上规避了病理分型所造成的误差，其准确性尚待进一步论证。

目前由于检测方法的不断问世，使得血液标志物的检测在肺癌筛查中越来越被关注，但作为单一筛查诊断手段，其敏感性和特异性仍不足以满足肺癌早期筛查的需要，尤其是目前对Ⅰ期肺癌不同直径结节诊断的敏感性和特异性研究已有初步结果^[36]^。但如何将液体活检与LDCT筛查结果相结合，提高肺癌早期筛查和诊断的敏感性和特异性，一直是国内外专家所关注的。

#### 3.2.2 循环肿瘤细胞（circulating tumor cell, CTC）

CTC是循环中自由存在的恶性肿瘤细胞，从原发肿瘤或转移部位脱离进入血液。近年来，新的技术已发展至可从外周血中识别、分离和鉴定这些循环肿瘤细胞。He等^[22]^在小鼠背部移植M109肺癌细胞，并分别在移植2周、3周和4周后，在小鼠耳部血管中每分钟检测到大约1.4个、7个和18个CTC细胞，说明随着肿瘤逐渐增大，CTC的数量呈指数级别上升。但在肿瘤移植的前4周内，没有在任何组织切片中发现存在转移性癌症的迹象。因此，理论上讲，在肿瘤发生的早期，循环中即可检测出CTC。有研究^[23-25]^结果表明，CTC与肿瘤分期密切相关，对肺癌诊断具有潜在的应用价值。目前已有多项研究^[26-28]^报道，通过叶酸受体（folate receptor, FR）靶向PCR CTC检测技术，检测非小细胞肺癌（non-small cell lung cancer, NSCLC）患者、肺部良性疾病患者和健康志愿者外周血中CTC数量，分析受试者工作特征曲线（receiver operating characteristic curve，ROC曲线）发现，当选择cutoff值为8.70 FU/3 mL时，诊断肺癌的敏感度为79.6%，特异度为88.2%，肺癌患者与非肺癌患者间CTC水平存在显著差异（*P*＜0.000, 1）。而且肺癌患者CTC水平与TNM分期相关，Ⅰ期NSCLC患者的诊断灵敏度达到67.2%，Ⅱ期、Ⅲ期、Ⅳ期患者阳性检出率分别为69.4%、80.9%和100%。而且与一般肿瘤标志物（CEA、NSE、CYFRA21-1、SCC-Ag）相比，基于FR靶向PCR的CTC检测方法具有更大的ROC面积（*P*＜0.000, 1），明显优于目前常用的一般肿瘤标志物。基于这些研究结果，叶酸受体阳性CTC检测试剂盒已经获中国国家食品药品监督管理局（China Food and Drug Administration, CFDA）批准，成为第一个上市的检测CTC的试剂盒。

#### 3.2.3 肺癌自身抗体

肺癌自身抗体的研究也受到关注。随着检测技术的进步，自身抗体的应用价值也不断被挖掘。研究发现在肿瘤发病早期，机体的免疫系统就可识别肿瘤细胞内表达异常的蛋白，引发免疫反应，由免疫生物信号放大系统产生大量抗体。而与其他血液分子标志物相比，肿瘤自身抗体有其独特的优越性：①早期灵敏度高：在癌症发生早期，由于抗原-抗体反应的高特异性和高敏感性，即使在血清中有大量白蛋白和其他蛋白的干扰，也可以在极低的浓度下被准确检测到；②高特异性：健康人与肺部良性病变者的血清中没有或含量很低，而肿瘤患者体内表达量提高，易于肿瘤早期鉴别诊断。单独的自身抗体分子特异性和敏感性都较低，无法满足肺癌早期筛查诊断的需要，而如果把若干自身抗体分子组合起来，进行联合筛查，可极大提高诊断的特异性和敏感性^[29]^。早在2008年，Chapman等^[30]^使用酶联免疫吸附实验（enzyme linked immunosorbent assay, ELISA）对104例肺癌患者与50例正常人群血清中7种肿瘤自身抗体（P53、c-myc、HER2、NYESO-1、GAGE、MUG1和GBU4-5）进行检测，发现单一抗体阳性率在5%-36%，诊断的特异性为96%-100%，而7种抗体联合检测的灵敏度为76%，特异性为92%，大大提高了肺癌的检出率。Boyle等^[31]^对6种肿瘤自身抗体（P53、NY-ESO-1、GAGE、GBU4-5、Annexin 1和SOX2）进行了临床检测，并将所研究病例依据肺癌病理类型和临床分期等分为三组，结果发现三组病例自身抗体诊断的灵敏度和特异性分别为36%和91%、39%和89%、37%和90%。这一结果为肺癌高危人群的筛查提供了可行的检测系统，这6种自身抗体的检测组合称为EarlyCDT-Lung，在随后的研究中发现将上述6种组合替换为7种（P53、NY-ESO-1、GAGE、GBU4-5、SOX2、HuD和MAGE A4）后，检测敏感性及特异性有显著的提高^[32]^。Jett等^[33]^对1, 613例肺癌高危人群进行了大规模的EarlyCDT-Lung检测，结果在最终诊断为肺癌的61例患者中有25例EarlyCDT-Lung检测阳性（灵敏度41%），并且在EarlyCDT-Lung检测阳性的肺癌患者中，57%为Ⅰ期或Ⅱ期。95%自身抗体检测结果阳性且伴有CT改变的患者是肺癌。目前肺癌自身抗体谱在北美已经临床应用，英国国家医疗服务体系（National Health Service, NHS）在苏格兰从2012年已开始全球第一个基于血清标志物的大型肺癌筛查项目，是一项血液肺癌筛查的里程碑式研究^[34]^。该研究是全球最大的用血液标志物进行筛查的研究，以7种肺癌自身抗体在肺癌高危人群中进行筛查，跟踪随访超过6年。2015年在世界肺癌大会（World Conference on Lung Cancer, WCLC）上公布了初步结果，肺癌筛查特异性91%，敏感性81%。2015年美国胸科协会（American Thoracic Society, ATS）公布的一组数据显示^[35]^，7种自身抗体技术单个自身抗体的特异性在96%以上，而两个自身抗体同时为阳性，特异性达99%。在另一项2015年ATS公布的临床研究中^[36]^，对美国296例肺小结节患者从2009年-2012年进行跟踪随访，发现7种自身抗体谱针对4 mm-20 mm的LDCT筛查后肺小结节检测阳性准确率达97%。七种肺癌自身抗体检测可以检测到有病灶的癌症与癌前病变，可以在症状出现之前对肺癌进行早期诊断，在筛查过程中定义谁需要进行肺癌筛查^[37, 38]^。我国在周彩存教授等专家的牵头下，开展了肺癌患者中7种肺癌自身抗体的检测（ELISA法）^[39]^。其中纳入肺癌患者818例，并以肺部良性疾病和健康人群为对照，结果显示肺癌组抗体浓度高于肺部良性疾病组（*P*＜0.01），总体敏感性达61%，特异性达90%。而且与传统的肿瘤标志物相比较，自身抗体的检测在Ⅰ期和Ⅱ期肺癌患者中具有很高的敏感性（62%和59%）。在该研究583例肺癌自身抗体阳性的人群中，CT影像学阳性523例，病理确认为肺癌497例，肺癌自身抗体联合CT检测肺癌阳性准确率95%，大大超过了单纯CT检查69%的阳性预测值。该项研究证实肺癌自身抗体谱的检测对肺癌不同分期尤其是Ⅰ期和Ⅱ期有很好的敏感性，有效弥补了临床上传统血清类标志物Ⅰ期与Ⅱ期敏感性不足的缺陷；同时具有良好的特异性，能较好地区分肺癌与良性疾病；而且与CT结合有很好的准确性，能辅助LDCT提高阳性预测值。鉴于此项研究结果，CFDA已于2015年11月批准上市7种肺癌自身抗体检测试剂盒。

目前由于检测方法的不断问世，使得血液标志物的检测在肺癌筛查中越来越被关注，但作为单一筛查诊断手段，其敏感性和特异性仍不足以满足肺癌早期筛查的需要，尤其是目前对Ⅰ期肺癌不同直径结节诊断的敏感性和特异性研究已有初步结果^[36]^。但如何将液体活检与LDCT筛查结果相结合，提高肺癌早期筛查和诊断的敏感性和特异性，一直是国内外专家所关注的。

## 4 展望

目前尚无大家公认的肺癌早期筛查的最佳模式。LDCT肺癌筛查的阳性结果已经拉开了建立肺癌早期筛查模式的大幕。但LDCT肺癌筛查中出现的高假阳性率、过度诊断和治疗、辐射暴露、经济费用等问题也使LDCT肺癌早期筛查倍受争议。肺癌血液筛查已显示出良好的敏感性和特异性，不论是国外的EarlyCDT-Lung自身抗体试剂盒还是我国目前已经批准上市的7种自身抗体试剂盒，都显示出与CT联合检查可以提高肺癌诊断的阳性预测值。高危人群的确定、LDCT肺癌筛查、血液筛查将有可能是肺癌早期筛查模式的三个重要组成部分。如何量化高危人群、如何将LDCT筛查和血液相关标志物检测有效结合，制定经济合理的筛查策略，提高肺癌早期检出率，降低假阳性率，避免过度诊治，建立肺癌筛查综合模式，尚需进行大规模的临床前瞻性研究。未来任重道远，我们期待建立更高效、更经济的肺癌筛查模式，真正实现肺癌的早期诊断。
